# Implantable starch-based extrudates for controlled release of corticosteroids

**DOI:** 10.1016/j.ijpx.2026.100625

**Published:** 2026-07-29

**Authors:** Paulo Vitor França Lemos, Julian Marcel Kunert, Marie-Luise Trutschel, Karsten Mäder

**Affiliations:** Institute of Pharmacy, Martin Luther University Halle-Wittenberg, Kurt-Mothes-Straße 3, 06120 Halle (Saale), Germany

**Keywords:** Corticosteroids, Local delivery, Postoperative inflammation, Long acting, Implants

## Abstract

Effective management of postoperative pain remains a major clinical challenge, driving the development of biodegradable implantable systems for localized drug delivery. Local control of postoperative inflammation plays an important role in pain management and represents a promising strategy for improving postoperative recovery. Starch-based implantable systems have recently emerged as promising biodegradable platforms for this purpose. Although polyester-based implants such as PLA and PLGA are widely used for sustained drug release, they present limitations, including poor mechanical behavior, delayed drug release, and the formation of acidic microenvironments during polymer degradation. In contrast, starch-based matrices represent an attractive alternative due to their intrinsic hydrophilicity, rapid hydration, and degradation into non-acidic products. This study aimed to characterize starch-based extrudates containing dexamethasone derivatives for potential application in postoperative pain and inflammation. Flexible cylindrical extrudates with nominal diameters of 600 μm and 1000 μm were produced using approved pharmaceutical excipients. Hydration studies revealed rapid water uptake within the first hour of incubation and swelling of approximately 30% without structural disintegration. Cyclodextrin-containing extrudates exhibited early matrix erosion within the first 24 h due to cyclodextrin dissolution, as confirmed by ^1^H NMR. Low-field ^1^H NMR relaxometry further revealed hydration-induced molecular reorganization within the starch matrix, with extrudates showing reduced chain mobility after incubation. Apparent solubility studies demonstrated a concentration-dependent increase in dexamethasone and dexamethasone acetate solubility in PBS in the presence of cyclodextrin. Drug release was primarily governed by extrudate diameter. Extrudates with 600 μm diameters showed faster release and reached approximately 80% cumulative release for dexamethasone and 60% for dexamethasone acetate within 10 days. Overall, starch-based extrudates enabled rapid hydration and sustained corticosteroid release for localized therapy.

## Highlights

• Flexible starch-based cylindrical extrudates with diameters of 600 μm or 1000 μm
• β-cyclodextrin acts as a matrix modifier
• Hydration induces molecular reorganization
• Extrudate diameter and β-cyclodextrin jointly modulate corticosteroid release
• Release kinetics appropriate for potential clinical applications

## Graphical abstract

**Figure:**
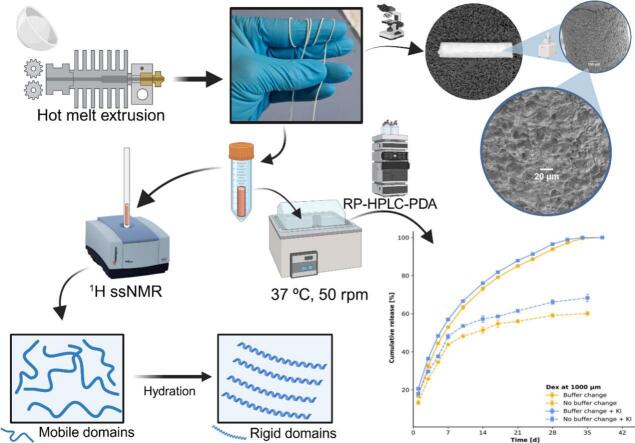

## 1 Introduction

Pain and inflammation are key features of numerous pathological conditions, including chronic inflammatory disorders and tissue injury following surgery. In the postoperative setting, inadequate control of these processes is associated with delayed functional recovery, impaired wound healing, prolonged hospital stays, increased risk of postoperative complications, and reduced patient quality of life (Chou et al., 2016; Kehlet and Dahl, 2003). Despite advances in surgical techniques and perioperative care, postoperative pain remains a major clinical challenge. A substantial proportion of patients still experience moderate to severe pain after surgery, which may hinder recovery and increase the risk of developing chronic pain (Kehlet and Dahl, 2003).

The need for improved therapeutic strategies has become increasingly important due to the substantial reliance on opioids for postoperative pain management (Chou et al., 2016). Although opioids remain essential for the treatment of moderate to severe acute pain, their use is associated with a broad spectrum of adverse effects, including respiratory depression, gastrointestinal disturbances, sedation, tolerance, and dependence (Volkow and McLellan, 2016). Recent clinical studies and systematic reviews have shown that a significant proportion of opioid-naïve surgical patients continue opioid use beyond the expected duration of acute postoperative pain, a phenomenon referred to as new persistent opioid use (Gong et al., 2024; Howard et al., 2023; Bicket et al., 2022). The prevalence of prolonged postoperative opioid use ranges from approximately 1% to 10%, depending on the type of surgical procedure, prescribing practices, and patient-specific factors (Gong et al., 2024; Howard et al., 2023; Volkow and Blanco, 2023). Prolonged opioid use after surgery not only adversely affects individual patients but also imposes a considerable socioeconomic burden through increased healthcare costs, hospital readmissions, long-term disability, and societal costs associated with opioid misuse and dependence (Volkow and Blanco, 2023). In response to these concerns, contemporary clinical guidelines strongly advocate multimodal analgesia strategies that combine agents with complementary mechanisms of action to reduce opioid dose and duration while maintaining effective pain control (Chou et al., 2016).

Dexamethasone is a potent synthetic glucocorticoid widely used to control inflammatory responses (Barnes, 2011). Its anti-inflammatory activity is primarily mediated through inhibition of phospholipase A₂ and modulation of transcription factors such as NF-κB and AP-1 (Barnes, 2011). In the postoperative setting, dexamethasone reduces peripheral inflammation and opioid requirements and is therefore widely incorporated into multimodal analgesia protocols (Chou et al., 2016).

Parenteral drug delivery systems, particularly implantable formulations, offer distinct advantages for treating localized inflammation and pain by enabling sustained and site-specific drug release (Fan et al., 2021). A prominent example is the FDA-approved intravitreal implant Ozurdex® (Abbvie, North Chicago, IL), which demonstrates the clinical feasibility of sustained dexamethasone delivery from biodegradable polymeric matrices (Lowder et al., 2011). However, Ozurdex® was developed for ocular indications and is not intended for postoperative applications.

Several implantable platforms for sustained dexamethasone delivery have been developed, predominantly based on PLGA. Hot-melt extruded PLGA implants provide prolonged local drug release, while implant geometry, polymer composition, and processing conditions can be tailored to modulate release kinetics (Fan et al., 2021; Lehner et al., 2019; Wachowiak et al., 2023). Injectable in situ forming PLGA depots have also been explored as an alternative strategy (Bode et al., 2018; Kempe and Mäder, 2012). Despite their widespread use in implantable drug delivery systems (Kempe and Mäder, 2012; Siepmann and Göpferich, 2001), PLA- and PLGA-based systems exhibit inherent limitations, including limited mechanical robustness, delayed drug release associated with lag phases, and the formation of acidic microenvironments during polymer degradation, which may compromise drug stability and release predictability (Ding and Schwendeman, 2008; Lehner et al., 2019; Mäder et al., 1996; Schädlich et al., 2014; Yang et al., 2024). Nevertheless, polyester-based systems remain the most widely used implantable platforms.

In contrast, starch-based implants offer several compelling advantages. Starch is an abundant semicrystalline polysaccharide composed of D-glucopyranose units with variable amylose content (Lemos et al., 2018; Lemos et al., 2019; Lemos et al., 2020; Lemos et al., 2021) that degrades into non-toxic and non-acidic products (Araújo et al., 2004). Previous studies by Esfahani and co-workers established starch-based extrudates as effective carriers for local drug delivery, demonstrating sustained and predictable drug release both *in vitro* (Esfahani et al., 2022; Esfahani et al., 2023a) and *in vivo* (Esfahani et al., 2023b). These systems successfully incorporated compounds with diverse physicochemical properties, including antimalarial drugs (Esfahani et al., 2022), the poorly water-soluble drug nimodipine (Esfahani et al., 2023a), and model fluorescent probes, while maintaining the mechanical integrity of the implants and showing good in vivo biocompatibility. Collectively, these findings support starch as a robust and versatile biopolymer for implantable drug delivery systems and provide a strong foundation for further formulation development.

The present study aims to explore the feasibility of developing dexamethasone-loaded implantable extrudates with high translational and industrial relevance that can be manufactured. In the present study, high-amylose starch and β-cyclodextrin, both generally recognized as safe (GRAS), were selected as matrix components to support regulatory acceptability and clinical translation. The extrudates were designed for subcutaneous implantation in postoperative settings requiring local control of inflammation and pain. Their biodegradable properties allow the implants to degrade in place without requiring surgical removal after drug depletion, while the localized and sustained corticosteroid delivery is intended to minimize systemic exposure. Hot-melt extrusion was employed as a robust and scalable pharmaceutical manufacturing technique widely used to produce flexible and ductile extrudates with therapeutically relevant drug loading and release. This approach aims to ensure reproducible manufacturing, preserve dexamethasone stability during extrusion and sterilization, and provide sufficient mechanical integrity for clinical handling. For the first time, the combined influence of cylindrical extrudate diameter and β-cyclodextrin (Kleptose®) incorporation on the release kinetics of dexamethasone and dexamethasone acetate from high-amylose corn starch (AMYLO N-400®) matrices was investigated. In addition, a formulation strategy is presented in which cyclodextrin acts as a matrix modifier to markedly enhance the apparent solubility of poorly water-soluble drugs. This approach enables starch-based extrudates to function as effective implantable depot systems for corticosteroid delivery.

## 2 Materials and methods

### 2.1 Material

High-amylose maize starch (AMYLO N-400®, 64.32 ± 0.64% amylose (ISO, 2007), CAS: 9005-25-8) and Kleptose® (β-cyclodextrin, CAS: 7585-39-9) were kindly provided by Roquette (Lestrem, France). Micronized dexamethasone (Euro OTC Pharma) and dexamethasone acetate (Caelo) were purchased and used as received. Dulbecco’s phosphate-buffered saline (DPBS) was purchased from Sigma-Aldrich (Munich, Germany). Acetonitrile and dimethyl sulfoxide (DMSO) were of chromatographic grade.

AMYLO N-400®, Kleptose®, dexamethasone, and dexamethasone acetate are hereafter referred to as Aml, Kl, Dex, and DAc, respectively.

### 2.2 Methods

#### 2.2.1 Extrudate preparation

Before extrusion, Aml and Kl were hydrated by adding two parts of the solid components to one part of water. The components were placed in a 50 mL Falcon tube and gently mixed manually using a spatula until a homogeneous mixture was obtained, with no visible agglomerates or material adhering to the tube walls. The mixture was then allowed to rest for 24 h. After this period, dexamethasone or dexamethasone acetate was added to obtain the different formulations. The final formulations were prepared as shown in Table 1.

**Table 1:** Composition of the starch-based extrudate formulations.

Formulation	Amylo N-400® (%)	Kleptose® (%)	Dexamethasone (%)	Dexamethasone acetate (%)	Nominal diameter (μm)
Aml	100	0	0	0	600 or 1000
Aml-Kl	91	9	0	0	600 or 1000
Aml-Kl-Dex	82	9	9	0	600 or 1000
Aml-Dex	82	9	9	0	600 or 1000
Aml-Kl-DAc	82	9	0	9	600 or 1000
Aml-DAc	82	9	0	9	600 or 1000

Extrudates were prepared by hot-melt extrusion (HME) using a ZE 5 ECO extruder (Three-Tec GmbH, Seon, Switzerland), based on the method described by Esfahani et al., 2023a with minor modifications. The temperature settings for the heating zones were 65 °C, 75 °C, and 85 °C, and the screw speed was set to 140 rpm. Extrusion dies with nominal diameters of 600 or 1000 μm were used to obtain cylindrical extrudates. The extrudates were collected and stored in Falcon tubes sealed with Parafilm® at 4–8 °C until further analysis.

#### 2.2.2 Electron beam sterilization

The extrudates were irradiated using a 10 MeV linear accelerator MB 10−30 MP (Mevex, Stittsville, Ontario, Canada) on a moving tray (95 cm min^-1^). The total sterilization dose of 25 kGy was delivered in two fractions of 12.5 kGy each (beam current 250 mA, PPS = 450 Hz), as previously described by Esfahani et al., 2023a.

#### 2.2.3 Extrudate characterization

##### 2.2.3.1 Optical and scanning electron microscopy

Optical microscopy was used to assess the homogeneity and diameter of the extrudates. Microscopic imaging was performed using an Olympus DSX1000 digital microscope (Tokyo, Japan) equipped with a super long working distance 3× objective lens (Model: DSX10-SXLOB3X). The microscope was operated in mixed mode, combining brightfield and darkfield illumination to enhance surface features. Samples were placed on the matte black rotary stage. No additional sample preparation was required before imaging. Image acquisition was carried out using the integrated software suite by Olympus, with constant lighting intensity and angle for all samples. Final image composition, as well as brightness, contrast, and saturation adjustments, were carried out using Pixelmator Pro (Apple Inc., Cupertino, CA).

Scanning electron microscopy (SEM) images were acquired using a Gemini 500 system (Zeiss Microscopy GmbH) under high vacuum. The accelerating voltage was set to 1 kV (EHT = 1.00 kV), and imaging was performed using secondary electrons to obtain high-resolution surface and cross-sectional images. Before imaging, the extrudates were frozen at -80 °C and gently cut using scalpels.

#### 2.2.4 Fourier-transform infrared spectroscopy with attenuated total reflectance

Infrared spectra of the excipients, drugs, physical mixtures, and extrudates were recorded using a Fourier-transform infrared (FTIR) spectrometer (Bruker Tensor IF37) equipped with an attenuated total reflectance (ATR) module containing a ZnSe crystal. Spectra were collected at room temperature (25 °C) over the range of 4000–400 cm^-1^. For each measurement, 32 scans were recorded at a spectral resolution of 4 cm^-1^.

#### 2.2.5 X-ray powder diffraction

X-ray powder diffraction (XRPD) experiments were performed using a D2-Phaser powder diffractometer (Bruker) operating in Bragg–Brentano geometry and equipped with a LYNXEYE XE-T strip detector. The instrument was fitted with a copper anode (30 kV, 10 mA) and Cu K_α_ radiation (λ = 0.15406 nm). Data were collected from rotating samples over a 2θ range of 8–35° with an angular resolution of 0.02° and a total acquisition time of 0.5 h (Lehner et al., 2025). The diffraction patterns were processed using the Bruker DIFFRAC.EVA software package (version 7.1). Powder samples were prepared as thin films on low-background sample holders made of oriented single-crystal silicon. Extruded samples were cut into smaller pieces of 1–3 mm in length before measurement.

#### 2.2.6 Thermogravimetric analysis

Thermogravimetric analysis (TGA) was carried out using a TGA/SDTA 851 instrument (Mettler-Toledo, Switzerland) equipped with an automatic sample changer (TS0801 RO). Approximately 5 mg of each sample was placed in 70 μL alumina crucibles and analyzed under a dynamic air atmosphere with a flow rate of 20 mL min^-1^. The thermal program consisted of an initial isothermal step at 25 °C for 5 min, followed by heating from 25 °C to 800 °C at a rate of 10 °C min^-1^ and a final isothermal step at 800 °C for 10 min. Mass loss and derivative thermogravimetric (DTG) curves were calculated using Origin 8.5 software.

#### 2.2.7 Differential scanning calorimetry

DSC thermograms were recorded using a DSC 823e module (Mettler Toledo, Gießen, Germany) with standard aluminum pans. Each sample was initially held at 25 °C for 2 min, heated to 90 °C at a rate of 10 °C min^-1^, and maintained at this temperature for 1 min. The samples were then cooled to -20 °C, held for 1 min, and reheated to 90 °C at the same heating rate under a nitrogen flow of 20 mL min^-1^. Data acquisition and processing were performed using STARe software (version 15.00, Mettler Toledo).

#### 2.2.8 Mechanical properties

Mechanical properties of the extrudates were determined according to the ISO 5079 standard method for textile fibers. Measurements were performed using a CT3-4500 Texture Analyzer (Brookfield Engineering, Middleboro, MA, USA). Samples approximately 30 mm in length were fixed between two padded clamps with a gauge length of 20 mm. The upper clamp was moved away from the lower clamp at a crosshead speed of 0.50 mm s^-1^. The force (F) was continuously recorded until sample failure occurred near the center. If breakage occurred at the clamp, the measurement was discarded and repeated with a new sample.

#### 2.2.9 Water uptake, matrix erosion, and swelling assays

Water uptake, matrix erosion, and swelling of the starch-based extrudates were evaluated according to the method described by Lehner et al., 2025, with adaptations to the starch-based extrudates. Cylindrical extrudate segments (approximately 1 cm in length) were weighed in the dry state (m_0_) and incubated in Falcon tubes containing 15 mL of phosphate-buffered saline (PBS, pH 7.4). Samples were maintained at 37 °C under gentle orbital agitation (25 rpm).

Five independent replicates of each formulation were analyzed. At predetermined time points (0, 1 h, 1, 3, and 7 days), the extrudates were carefully removed, gently blotted with lint-free tissue to remove surface moisture, and weighed to determine the wet mass (m_wet_). The samples were then dried in a forced-air oven at 40 °C until constant weight (m_dry (t)_).

Water uptake and mass remaining were calculated using Equations (1), (2):

(1) $$\text{Water uptake}\;\left(\%\right)=\frac{m_{\mathrm{wet}}-m_{\mathrm{dry}}}{m_{\mathrm{wet}}}\times 100$$

(2) $$\text{Mass remaining}\;\left(\%\right)=\frac{m_{\mathrm{dry}}\left(t\right)}{m_{\mathrm{dry}}\left(0\right)}\times 100$$

Swelling was determined from micrographs of the hydrated extrudates acquired at each time point (0, 1 hour, 3 hours, 2 days, 3 days, and 7 days). The swelling degree was calculated based on the change in apparent area (A) according to equation (3):

(3) $$\text{Swelling degree}\;\left(\%\right)=\frac{A_{t}-A_{0}}{A_{0}}\times 100$$

where *A₀* and *Aₜ* correspond to the initial and time-dependent cross-sectional areas, respectively.

The PBS medium was replaced after each sampling time point to maintain a constant ionic strength and pH. All measurements were performed in five replicates (n = 5), and results are presented as mean ± standard deviation.

#### 2.2.10 ^1^H NMR spectroscopy

Aml and Aml-Kl extrudates were analyzed by ^1^H NMR spectroscopy to evaluate the release of β-cyclodextrin into the aqueous medium. A reference solution of Kleptose® was prepared by dissolving the powder directly in D_2_O at a concentration of 14 mg mL^-1^.

For release experiments, Aml and Aml-Kl extrudates (1 cm in length, approximately 10 mg per piece) were incubated in 1 mL of 0.09% (w/v) NaCl solution prepared in D_2_O. Incubations were carried out in an orbital shaker at 37 °C and 50 rpm. After incubation, samples were centrifuged (3000 × g, 10 min, 20 °C) to remove solid residues, and the resulting supernatants were collected for analysis.

^1^H NMR spectra were recorded on an Agilent Technologies VNMRS spectrometer operating at 400 MHz using a 5 mm probe equipped with pulsed field gradients. Data acquisition and processing were performed using VNMRJ 3.2 software. Chemical shifts (δ) are reported in parts per million (ppm) and referenced to the residual HOD signal.

#### 2.2.11 Relaxometry assays

Relaxometry experiments were performed following the general approach described by Esfahani et al., 2023a using solid-state ^1^H nuclear magnetic resonance. Extrudates were cut into 1 cm pieces and placed in 10 mm NMR tubes. The transverse relaxation time (T_2_) was determined using a Bruker Minispec mq20 relaxometer operating at 20 MHz and 37 °C. Measurements were conducted in the dry state and after hydration.

For hydrated samples, 1 mL of 0.9% (w/v) NaCl solution prepared in D_2_O was added to the tube, and the samples were incubated for 1 h. After removing excess solution, T_2_ was measured again under identical conditions.

The magic sandwich echo (MSE) pulse sequence was employed to minimize dead-time effects and enable reliable quantification of magnetization decay in rigid and mobile domains. The MSE sequence was applied during the initial microseconds of acquisition to recover rapidly decaying signal components, after which the free induction decay (FID) was recorded to monitor the subsequent transverse magnetization decay. The resulting magnetization decay curves were fitted to bi-exponential models according to Equation (4).

(4) $$y=A_{1}\exp\left(-x/T_{2,1}\right)+A_{2}\exp\left(-x/T_{2,2}\right)+y_{0}$$

where *y* is the signal amplitude at time *x*, *A*_*1*_ and *A*_*2*_ represent the relative amplitudes (fractions) of proton populations exhibiting distinct relaxation behaviors, and *T*_*2,1*_ and *T*_*2,2*_ correspond to their respective transverse relaxation times. The baseline offset is represented by *y*_*0*_.

The distinct T_2_ components were interpreted as proton populations with different mobilities, corresponding to rigid (short *T*_*2,1*_) and mobile (long *T*_*2,2*_) domains within the polymeric matrix. Changes in *T*_*2*_ values and in the relative proportions of rigid and mobile components were used to infer the extent of water penetration and polymer chain mobility, thereby providing insight into the structural dynamics of the matrix.

#### 2.2.12 In vitro release assays

##### 2.2.12.1 Drug load, release, and recovery assays

To determine the drug load, extrudate segments (1 cm in length) were finely cut using a scalpel, weighed, and transferred to 20 mL of a DMSO:water mixture (90:10, v/v). A 1 cm extrudate corresponds to a nominal drug content of approximately 0.4 mg for the 600 μm formulations and approximately 1.0 mg for the 1000 μm formulations. The samples were stirred at 60 °C for 1 h and then allowed to cool to 25 °C while continuing to stir for 24 h. Subsequently, the solutions were diluted 1:5 with water:acetonitrile (50:50, v/v), filtered through a 0.22 μm PVDF syringe membrane filter, and stored at -18 °C until analysis.

For drug release evaluations, extrudate segments approximately 1 cm in length were gently cut and weighed. Each sample was placed in Falcon tubes containing either 15 mL or 50 mL of PBS (pH 7.4) for Dex and DAc, respectively, to account for their different solubilities. Incubation was performed at 37 °C and 50 rpm in a shaking incubator (Memmert GmbH + Co. KG, Schwabach, Germany) under light-protected conditions. At predetermined time points, the entire buffer volume was withdrawn, filtered through a 0.22 μm PVDF syringe membrane filter (Carl Roth GmbH + Co. KG, Karlsruhe, Germany), and stored at -18 °C until analysis. In parallel, no buffer change experiments were performed under identical conditions, except that the release medium was not replaced. At each sampling time point, a 100 μL aliquot was withdrawn, diluted with the chromatographic eluent to a final volume of 1 mL, filtered through a 0.22 μm PVDF syringe membrane filter, and stored for analysis, while the remaining medium was kept in the system.

After completion of the release assays, each extrudate was quantitatively removed from the Falcon tubes and subjected to the same procedure described for the drug load assay to determine the recovery of Dex and DAc. The recovered drug amount was used to determine the absolute drug content of each individual extrudate, with this amount defined as 100% release. The resulting release values were compared with those calculated using the nominal drug load, and both approaches yielded comparable results.

Drug load, release, and recovery samples were analyzed by reverse-phase chromatography using the same instrumental method. All experiments were conducted in three independent replicates (n = 3).

For the release studies of DAc-containing formulations, both DAc and Dex were quantified by RP-HPLC-PDA. The total amount of DAc released was calculated as the sum of the measured DAc concentration and the measured Dex concentration converted to its DAc equivalent using the respective molecular weights, according to Equation (5):

(5) cDAc,total=cDAc,measured+cDex,measured×434.50gmol392.47gmol

where *C*_*DAc,total*_ is the total DAc concentration used to construct the release profiles, *C*_*DAc,measured*_ is the DAc concentration directly quantified by RP-HPLC-PDA, *C*_*Dex,measured*_ is the Dex concentration directly quantified by RP-HPLC-PDA, and 434.50 and 392.47 g/mol correspond to the molecular weights of dexamethasone acetate and dexamethasone, respectively.

#### 2.2.13 Influence of Kleptose® on dexamethasone solubility

The apparent solubility of Dex and DAc was determined by equilibrium solubility (shake-flask) assays. Experiments were performed in PBS (pH 7.4) containing Kleptose® at concentrations of 0, 0.025, 0.050, 0.100, 0.250, and 0.500 mM. For Dex, assays were conducted in 15 mL Falcon tubes containing 15.0 mL of medium, whereas for DAc, assays were performed in 50 mL tubes containing 50.0 mL of medium. After complete dissolution of Kleptose®, an excess amount of Dex (∼30 mg) or DAc (∼30 mg) was added to each tube to ensure saturation conditions. The suspensions were incubated at 37 °C for 48 h under agitation (50 rpm) to allow equilibration between the solid and dissolved phases. After incubation, samples were centrifuged (3000 × g, 10 min, 20 °C), and the supernatants were carefully collected and filtered through 0.22 μm PVDF syringe filters. The filtered samples were analyzed by reverse-phase high-performance liquid chromatography (RP-HPLC). All experiments were performed in triplicate.

#### 2.2.14 Evaluation of dexamethasone acetate stability in PBS

DAc (0.758 mg) was accurately weighed and transferred into a beaker containing 100 mL of PBS. The dispersion was magnetically stirred at 300 rpm and 20 °C until complete dissolution was achieved. The solution was then filtered through 0.22 μm PVDF syringe filters using 10 mL syringes, with the filter replaced after every 10 mL. A 50 mL aliquot of the filtered solution was transferred to a 50 mL Falcon tube and incubated at 37 °C under agitation (50 rpm). Aliquots were collected daily, diluted 1:1 (v/v) with the HPLC mobile phase, and stored at -18 °C until chromatographic analysis.

#### 2.2.15 Reverse-phase high-performance liquid chromatography

A method for the simultaneous quantification of Dex and DAc was developed using a Waters^TM^ Arc HPLC system (Boston, MA, USA), comprising a Quaternary Solvent Manager-R, a Sample Manager FTN-R with an integrated column oven, and a 2998 photodiode array (PDA) detector. Isocratic elution was performed using a mobile phase of acetonitrile: water (50:50, v/v). Separation was achieved on a Phenomenex® Kinetex® Evo C18 column (150 mm × 4.6 mm, particle size 4.6 μm) with an injection volume of 20 μL, at a flow rate of 0.750 mL min^-1^ and a column temperature of 40 °C. Detection was carried out at a wavelength of 239 nm. The total run time was 10 min. Linear calibration curves were obtained for Dex (Rt = 2.6 min, R^2^ = 1.00) and DAc (Rt = 4.1 min, R^2^ = 0.99) over the concentration range of 2.5–100 μg mL^-1^.

## 3 Results and discussions

### 3.1 Preformulation evaluations

Preformulation studies were conducted to identify suitable starch-drug-cyclodextrin formulations. High-amylose starch (Amylo N-400®) was selected based on previous preformulation and extrusion studies from our research group, which demonstrated superior processability and the production of flexible extrudates with good structural integrity. β-Cyclodextrin (Kleptose®) was incorporated to enhance the apparent solubility of the corticosteroids while preserving the extrusion processability of the formulations. The screening strategy focused on the influence of Kleptose® content on the macroscopic properties of the extrudates before detailed structural, dimensional, and functional characterization.

Formulations were systematically evaluated by varying the Kleptose® content within the starch matrix, as summarized in **Table S1**. Extrudates containing 5–10% (m/m) cyclodextrin exhibited a homogeneous appearance and good flexibility, whereas higher cyclodextrin contents (≥ 20% m/m) produced brittle extrudates prone to fracture during handling. Based on this screening, cyclodextrin contents up to 10% (m/m) were selected as the optimal compromise between matrix modification and mechanical integrity. The ability to produce flexible and ductile extrudates is a key requirement for implantable systems intended for subcutaneous administration, as adequate mechanical robustness is essential for surgical handling, insertion through small incisions, and resistance to fracture during implantation.

Similar trends have been reported for starch-based extrudates, particularly the work of Esfahani et al., 2022, who emphasized the importance of formulation screening during the preformulation stage to obtain mechanically compliant starch extrudates suitable for biomedical applications. Similar to the present study, their results demonstrated that excessive matrix modification or disruption of starch–starch interactions led to brittle structures, highlighting the existence of a relatively narrow processing window in which flexibility and structural integrity can be simultaneously achieved.

The preformulation studies enabled the rational selection of the formulation composition that ensured adequate mechanical performance, handling robustness, and suitability for subsequent processing and structural characterization. Based on these results, formulations containing 9% (m/m) Kleptose® and 9% (m/m) dexamethasone derivatives were selected for further investigation, as this composition provided a suitable balance between matrix modification, drug loading, and mechanical integrity while maintaining good processability during extrusion.

### 3.2 Characterization of the extrudates

#### 3.2.1 Morphology

**Fig. 1:**
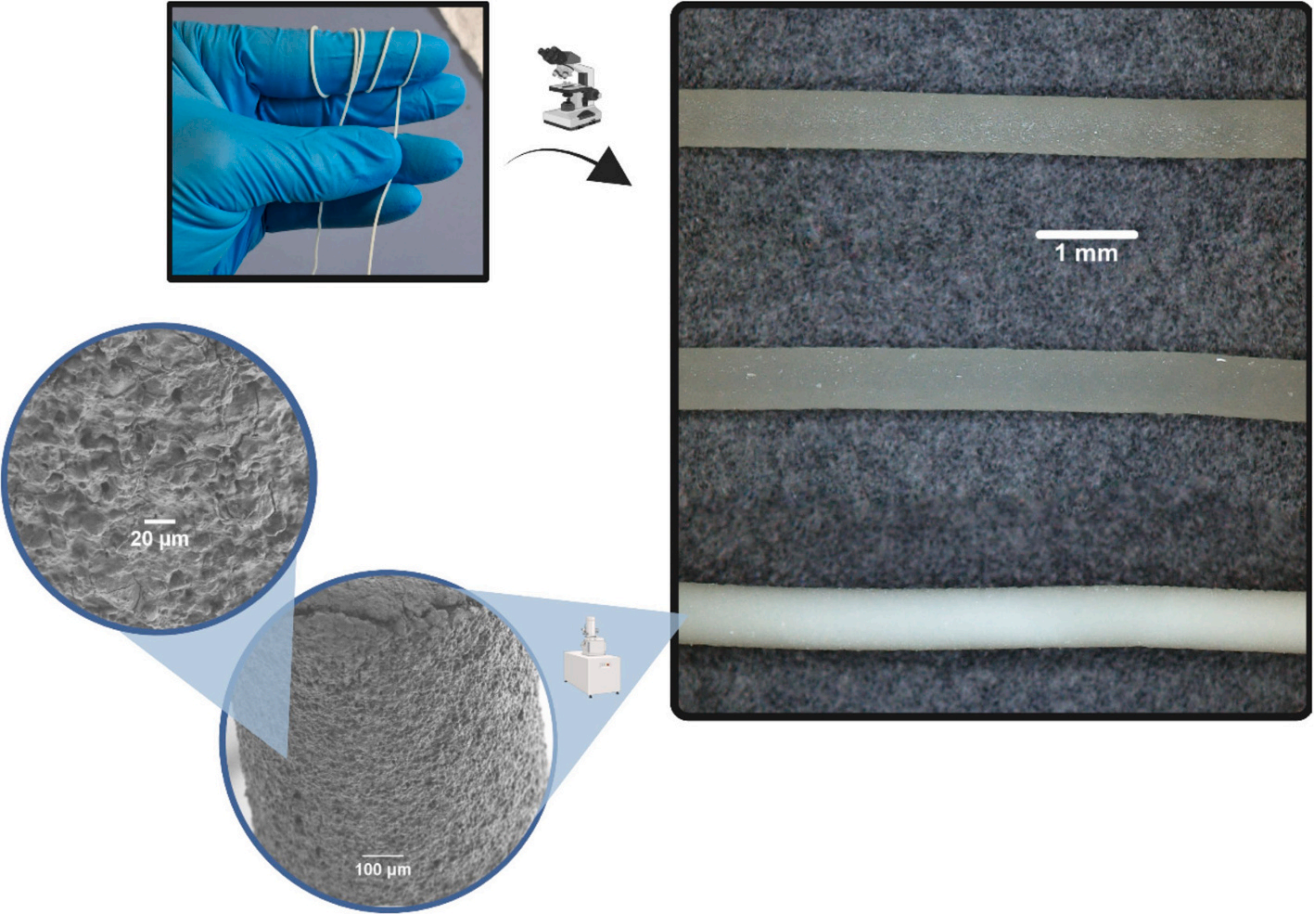
Representative optical and scanning electron microscopy micrographs of 600 μm starch-based extrudates Aml, Aml-Kl, and Aml-Kl-Dex (from top to bottom)

Fig. 1 presents representative optical and SEM images of the starch-based extrudates. The production of visually homogeneous cylindrical extrudates is further illustrated in **Figs. S1 (a) and (b)**. Independent extrusion batches consistently yielded extrudates with no visible macroscopic phase separation, indicating satisfactory process reproducibility. At the microscale, the extrudate surfaces exhibited a certain degree of roughness, reminiscent of residual starch granule structures with occasional small discontinuities. Extrudates with a diameter of 600 μm displayed a similar surface pattern, as shown in **Figs. S1 (c) and (d)**.

Partial gelatinization of native starch granules occurred during extrusion. This behavior can be attributed to the combined effects of moderate water content in the powder mixture (>30% m/m), elevated temperature during extrusion (85 °C), and applied shear forces. Under such thermomechanical conditions, extrusion primarily disrupts the outer regions of starch granules, while inner domains may remain partially intact, resulting in heterogeneous granular remnants within the extrudates (Huang et al., 2022; Li et al., 2014). Experimental extrusion studies further demonstrate that amylose can diffuse out of starch granules during processing, particularly under conditions of elevated specific mechanical energy, without undergoing extensive molecular degradation (Huang et al., 2022; Li et al., 2014; McPherson and Jane, 2000). The leached amylose contributes to an amorphous, solubilized fraction distributed between granular remnants and thereby promotes intergranular cohesion and structural continuity by filling voids between partially preserved granular structures (Huang et al., 2022; McPherson and Jane, 2000).

In addition to microstructural homogeneity, the dimensional characteristics of the extrudates are summarized in Table 2. Cylindrical extrudates with nominal diameters of 600 μm and 1000 μm were successfully prepared in multiple extrusion runs, although minor variations were observed for some formulations. The consistent production of extrudates with reproducible diameters demonstrates stable processing behavior under the applied extrusion conditions. Diameter uniformity is particularly relevant for implantable formulations, as the cross-sectional geometry directly governs the surface-area-to-volume ratio of the device, which is a key determinant of mass transport and drug release kinetics from subcutaneous implants (Domsta et al., 2023; Iyer et al., 2006), while also affecting the mechanical integrity and structural stability required to withstand handling and implantation without fracture or deformation.

**Table 2:** Diameters and mechanical properties of the 600 μm and 1000 μm extrudates.

Sample	Extrudates 600 μm				Extrudates 1000 μm			
	Diameter	Tensile strength (MPa)	Elongation at break (%)	Young's modulus (MPa)	Diameter	Tensile strength (MPa)	Elongation at break (%)	Young's modulus (MPa)
Aml	607.9 ± 13.3	22.79 ± 2.06	74.93 ± 0.03	717.4 ± 55.9	956.7 ± 8.1	14.8 ± 0.4	9.9 ± 0.2	396.2 ± 40.9
Aml-Kl	634.7 ± 6.0	15.30 ± 4.48	50.00 ± 0.00	556.6 ± 135.4	1025.0 ± 3.0	10.0 ± 2.3	5.0 ± 0.5	386.0 ± 23.3
Aml-Kl-Dex	655.6 ± 15.2	9.38 ± 1.89	49.98 ± 0.03	442.5 ± 63.7	967.1 ± 16.3	6.4± 1.1	2.7 ± 0.2	470.9 ± 36.3
Aml-Dex	607.4 ± 9.2	13.52 ± 2.45	49.97 ± 0.03	532.8 ± 39.5	1052.3 ± 17.4	5.4± 1.5	3.9 ± 0.3	320.7 ± 70.4
Aml-Kl-DAc	626.3 ± 16.6	14.03 ± 2.88	49.97 ± 0.03	641.5 ± 134.0	973.3 ± 28.7	8.8 ± 0.5	2.9 ± 0.2	457.4 ± 10.2
Aml-DAc	581.0 ± 14.2	17.25 ± 2.45	49.95 ± 0.00	730.2 ± 147.3	996.8 ± 23.5	8.0 ± 1.5	4.4 ± 0.3	436.3 ± 114.4

The extrudates exhibited macroscopic homogeneity and mechanical robustness. This behavior is structurally supported by the coexistence of partially preserved granular remnants, an amorphous binding phase, and controlled extrudate dimensions. Such morphological features are advantageous for implantable drug-delivery systems, as they promote uniform drug distribution within the matrix while maintaining sufficient cohesion and dimensional stability during handling and implantation.

Raman microscopy provided consistent evidence of content uniformity across the cross-sections of the starch-based extrudates (**Fig. S1 (d)**). Comparison between the control extrudates (Aml-Kl) and the drug-loaded samples (Aml-Kl-Dex) revealed highly consistent spectral profiles throughout the mapped regions, without localized increases in Raman intensity or spectral features indicative of drug-rich domains. The absence of spatially resolved variations in the Raman response indicates that neither micrometer-scale crystalline agglomerates nor compositional gradients were present within the analyzed cross-sections. At the micrometer scale probed by Raman mapping, these results provide clear evidence of a homogeneous distribution of dexamethasone within the extruded starch-based matrices.

Similar observations were reported by Esfahani et al., 2023a for starch-based extrudates loaded with nimodipine, where Raman microscopy confirmed a homogeneous drug distribution across the extrudate cross-sections. The extrusion process promotes efficient mixing and molecular-level dispersion of the drug within the starch matrix, supporting the suitability of extrusion-based processing for achieving content uniformity in starch-based controlled-release systems.

#### 3.2.2 Thermal properties and microstructure

The thermal properties of AMYLO N-400®, Kleptose®, Dex, and DAc were evaluated to assess their compatibility with hot-melt extrusion at a maximum processing temperature of 85 °C. All components exhibited thermal stability above 200 °C in thermogravimetric analysis, indicating a substantial safety margin relative to the applied extrusion conditions (**Fig. S2 (a)**). Differential scanning calorimetry revealed no thermal transitions, melting events, or exothermic phenomena up to 85 °C for either excipients or drugs (**Fig. S2 (b)**). The thermal data indicate a consistent and predictable thermal response of the powder mixture under the applied processing conditions.

The infrared spectra of the excipients (**Fig. S2 (c)**) displayed closely comparable profiles, consistent with their glycosidic chain structures (Lemos et al., 2018; Lemos et al., 2019; Lemos et al., 2020). The active pharmaceutical ingredients exhibited similar spectral features (**Fig. S2 (d)**) consistent with their respective molecular structures (Linstrom and Mallard, 2026a; Linstrom and Mallard, 2026b). The spectra of the physical mixtures containing the excipients or DAc closely matched those of the corresponding extrudates. No new absorption bands or band shifts were observed after extrusion (**Fig. S2 (e)**). These results indicate that the extrusion process did not induce detectable chemical modification or degradation of the formulation components.

XRD analysis revealed distinct solid-state characteristics for the individual formulation components. AMYLO N-400® exhibited a semicrystalline diffraction pattern with a dominant broad peak around 17.0° 2θ (**Fig. S2 (f)**), typically associated with retrograded amylose (Lemos et al., 2018; Lemos et al., 2019). Kleptose® showed a highly crystalline profile, characterized by a strong reflection ∼ 9.0° 2θ and a doublet at around 12.5° and 12.8° 2θ (**Fig. S2 (f)**). Dex displayed pronounced crystalline signatures, with a characteristic reflection at approximately 13.6° 2θ (**Fig. S2 (g)**), consistent with reported diffraction patterns of crystalline dexamethasone (Alami-Milani et al., 2018). DAc also exhibited a crystalline profile, with an intense low-angle reflection at approximately 10.0° 2θ (**Fig. S2 (g)**), in agreement with published XRD data for dexamethasone acetate (Bastos et al., 2023). These diffraction features reflect the intrinsic crystallinity of the drugs and cyclodextrin, as well as the semicrystalline nature of the starch matrix before processing.

The Aml-Kl extrudate retained the semicrystalline structure of the starch matrix after extrusion (**Fig. S2 (h)**). Characteristic crystalline reflections associated with Dex remained detectable in the corresponding drug-loaded formulations (**Fig. S2 (h)**). Similar reflections were also detected in DAc-containing extrudates (**Fig. S2 (h)**). These findings indicate that the applied thermomechanical conditions did not induce complete amorphization of the drug components. Accordingly, the extrudates represent semicrystalline systems dominated by starch- and dexamethasone-derived crystalline domains, with minimal contribution from cyclodextrin. The comparative diffraction profiles in **Fig. S2 (i)** illustrate this solid-state organization and its preservation after extrusion.

#### 3.2.3 Mechanical properties

Table 2 summarizes the mechanical properties of the starch-based extrudates processed using dies with nominal diameters of 600 μm and 1000 μm. A consistent mechanical trend was observed when comparing samples obtained from the two die diameters, regardless of formulation. In most cases, reducing the extrusion diameter resulted in increased tensile strength and Young’s modulus, while elongation at break also increased (Table 2).

The incorporation of crystalline components reduced the mechanical performance of the Aml extrudates. Tensile strength and elongation at break decreased in formulations containing Kleptose®, Dex, or DAc, regardless of extrusion diameter (Table 2). This mechanical weakening can be attributed to the presence of rigid crystalline domains that disrupt the continuity of the starch matrix and impair efficient stress distribution under load. This interpretation is supported by the compact morphology observed by SEM before incubation (**Fig. S1 (c)**), indicating that the crystalline additives were incorporated into a continuous starch matrix rather than forming large structural defects. Furthermore, DSC confirmed the absence of detectable thermal transitions within the extrusion temperature range, while XRD demonstrated the persistence of characteristic crystalline domains after processing (**Figs. S2 (b) and (h)**), suggesting that these rigid phases remained dispersed within the starch matrix and acted as a point of failure during tensile loading. Previous studies on starch-based blends have shown that poor integration of rigid phases reduces interfacial cohesion, thereby impairing stress transfer and resulting in lower tensile strength and ductility (Jayarathna et al., 2022).

Kleptose® reduced the mechanical flexibility of starch extrudates. Despite its hydrophilic and starch-related structure, it has been reported to crystallize within starch matrices, thereby lowering cohesion and flexibility (Jayarathna et al., 2022). Dex and DAc are crystalline and poorly miscible with starch, and hydrophobic corticosteroids commonly remain as crystalline phases in hydrophilic polymer matrices (Alami-Milani et al., 2018; Fan et al., 2021). Under such conditions, weak interfacial adhesion may promote phase separation and the formation of discrete crystalline inclusions that hinder stress transfer across the polymer–drug interface. Consequently, extrudates containing these crystalline components exhibited lower resistance to fracture and a reduced capacity for plastic deformation.

A comparable trend was observed for the elastic modulus (Table 2). The addition of crystalline components generally decreased the modulus, indicating reduced structural integrity and interchain stiffness (Aaliya et al., 2021). However, this effect was more pronounced and systematic in the 600 μm extrudates, whereas it was less evident in the 1000 μm samples (Table 2). In smaller-diameter extrudates, where deformation history and densification effects are expected to be more significant, the starch matrix may become more orientation-dependent in its mechanical response (Xie et al., 2012). Under these conditions, crystalline and poorly compatible particles can more strongly disrupt the load-bearing microstructure, leading to a more pronounced reduction in modulus.

Despite differences in mechanical properties, all formulations remained mechanically robust. Handling and manual manipulation did not result in visible fractures (**Figs. S1 (a) and (b)**). These observations indicate that extrusion produced cohesive starch-based matrices with sufficient load-bearing capacity, regardless of extrudate diameter. Overall, the mechanical data support the suitability of both extrudate sizes for further evaluation as biodegradable drug delivery systems, while highlighting extrudate diameter as a key parameter influencing mechanical performance.

#### 3.2.4 Water uptake, matrix erosion, and swelling assays

**Fig. 2:**
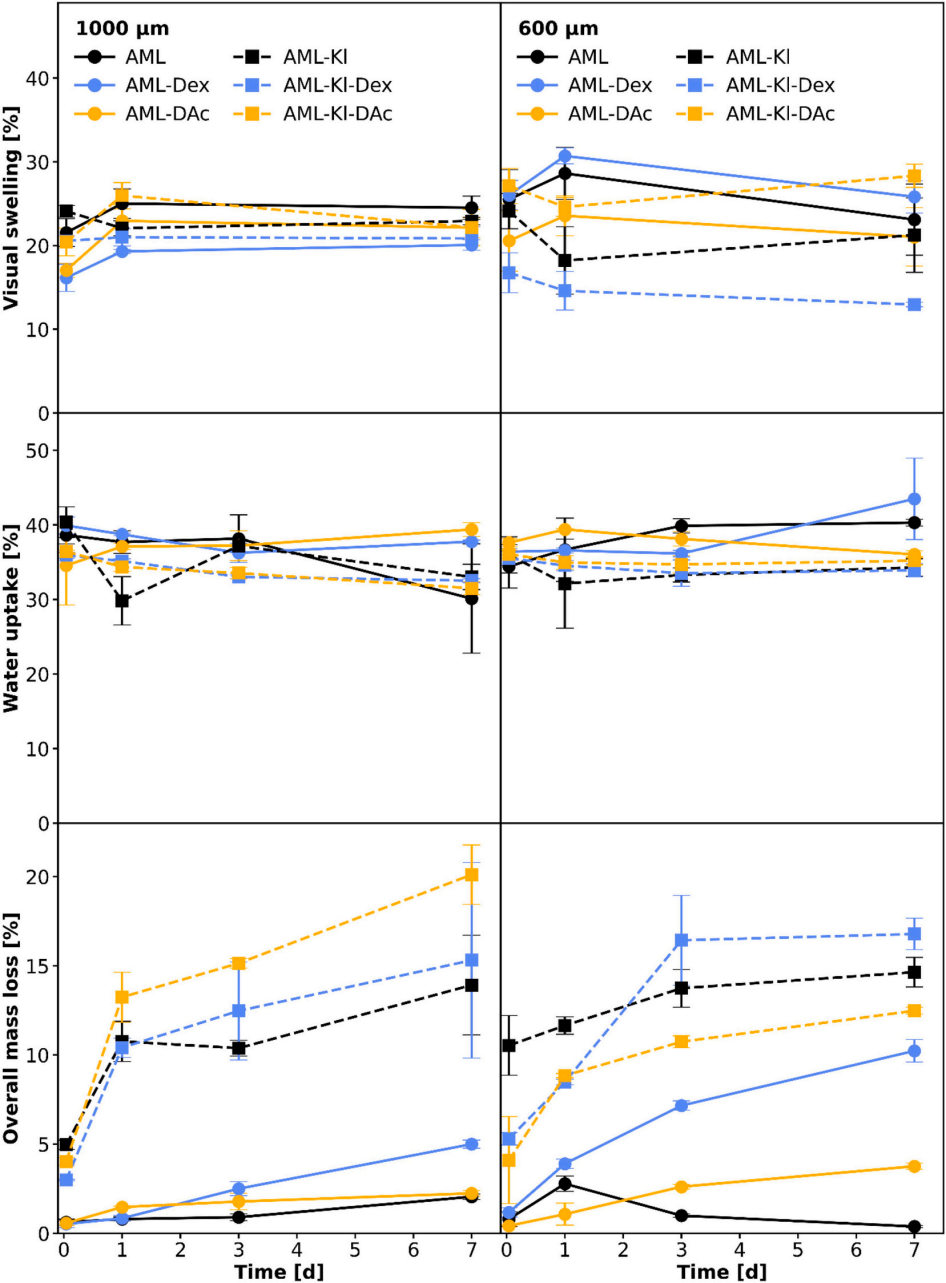
Swelling, water uptake, and matrix erosion of the extrudates incubated in PBS at 37 °C were measured after 1 hour, 1 day, 3 days, 5 days, and 7 days of incubation.

Water uptake, matrix erosion, and swelling of the starch-based extrudates are presented in Fig. 2. All formulations exhibited rapid hydration upon incubation in PBS at 37 °C, with a pronounced increase in water uptake already observed after 1 h. This initial hydration phase was followed by a more gradual increase, reaching quasi-plateau values between 3 and 7 days. No delayed hydration behavior was observed for any formulation.

Water uptake profiles were comparable for the 600 μm and 1000 μm extrudates (Fig. 2). The incorporation of Dex or DAc did not significantly affect the overall hydration behavior relative to the corresponding drug-free matrices.

Matrix erosion remained limited throughout the incubation period (Fig. 2). After 3 days, formulations containing Kleptose® (Aml-Kl, Aml-Kl-Dex, and Aml-Kl-DAc) exhibited a mass loss of approximately 12%, whereas extrudates without cyclodextrin (Aml, Aml-Dex, and Aml-DAc) showed substantially lower erosion levels. Beyond the third day, erosion profiles stabilized, indicating that mass loss occurred predominantly during the early stages of incubation.

Swelling followed trends closely aligned with water uptake for all formulations (Fig. 2). A rapid increase in extrudate swelling was observed during the initial hydration phase, followed by stabilization over time. No structural disintegration was detected, indicating that hydration-induced expansion remained around 30%.

Hydration of the starch-based extrudates occurred markedly faster than typically reported for polyester-based implant systems. In contrast to PLGA extrudates, which often exhibit limited early water uptake due to the hydrophobic nature of the polymer backbone, starch matrices absorbed water rapidly within the first hour of incubation. Lehner et al., 2025 and Bode et al., 2019 reported that PLGA-based dexamethasone implants show limited initial water uptake, leading to gradual hydrolysis of the polymer chains and subsequent swelling once a critical reduction in molecular weight is reached. The onset of swelling is typically observed after a lag phase lasting from days to weeks, during which little or no drug release occurs. In comparison, the immediate hydration observed for the starch-based extrudates reflects the intrinsic hydrophilicity of starch rather than matrix degradation, supporting their suitability for applications requiring early and predictable hydration-driven drug release. Compared to PLGA, this behaviour of starch represents an advantage for the release of hydrophobic drugs, but also a challenge for the controlled release of hydrophilic molecules.

Matrix erosion was formulation-dependent and strongly associated with the presence of Kleptose®. Extrudates containing cyclodextrin (Aml-Kl, Aml-Kl-Dex, and Aml-Kl-DAc) exhibited an early mass loss consistent with the nominal cyclodextrin content, whereas formulations without Kleptose® (Aml, Aml-Dex, and Aml-DAc) showed minimal erosion. These results indicate that the observed erosion predominantly reflects cyclodextrin dissolution rather than degradation of the starch backbone.

The early release of Kleptose® is independently supported by the ^1^H NMR spectra (**Fig. S3**). No proton signals were detected in the D_2_O extracts obtained from Aml extrudates, indicating that no soluble components were released from the AMYLO N-400® matrix. In contrast, the spectra of the Aml-Kl extrudates exhibited characteristic peaks that coincided with those of a reference solution of pure Kleptose®. Together, the gravimetric and spectroscopic data demonstrate that matrix erosion in these systems is a controlled, composition-driven process. Kleptose® is selectively released upon hydration, while the starch matrix remains cohesive without visible structural changes.

#### 3.2.5 NMR relaxometry assays

**Fig. 3:**
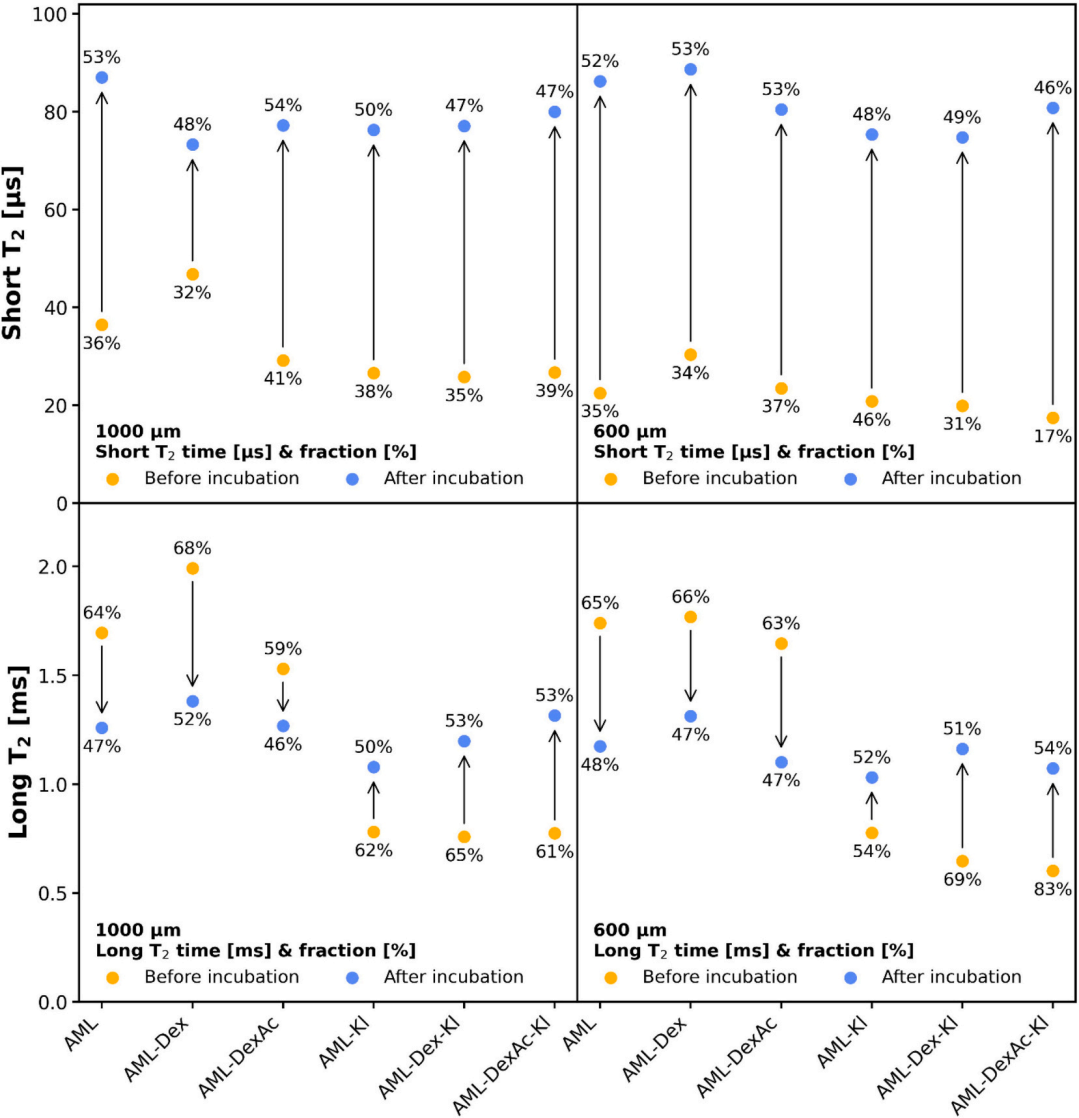
Short (T_2,1_) and long (T_2,2_) transverse relaxation components obtained from ^1^H NMR relaxometry of starch-based extrudates (600 μm and 1000 μm). Results are presented as relaxation times (μs and ms) and relative percentage contributions of each component. A comparison between dry (before incubation) and wet (after incubation in 0.9% NaCl solution in D_2_O for 1 h at 37 °C) states is shown.

Fig. 3 summarizes both transverse relaxation components obtained from the ^1^H NMR relaxometry assays of the 600 μm and 1000 μm extrudates, namely the short (T_2,1_) and long (T_2,2_) T_2_ contributions. The results are presented as relaxation times in milliseconds (ms) and as relative contributions of each component. While T_2,1_ reflects proton populations in rigid and highly constrained domains, T_2,2_ is associated with more mobile chain segments, enabling a comprehensive assessment of the distribution of molecular mobility within the starch-based matrices.

In multicomponent ^1^H NMR relaxometry, longer T_2_ components are typically associated with more mobile chain segments or loosely constrained proton environments, whereas shorter T_2_ values reflect increasingly restricted molecular motion within ordered domains (Adamczyk et al., 2025; El Nokab et al., 2022). In this context, T_2_ corresponds to the transverse relaxation time describing the decay of spin–spin coherence governed by dipolar interactions and modulated by local molecular dynamics.

The transverse magnetization decay curves were fitted using bi-exponential models to extract both short (T_2,1_) and long (T_2,2_) relaxation components and their relative contributions, as illustrated for the 600 μm and 1000 μm extrudates in **Figs. S4 (a-l)** and **S5 (a-l)**, respectively. This fitting procedure enabled the resolution of proton populations with distinct mobility, assigned to rigid (T_2,1_) and mobile (T_2,2_) domains within the starch-based matrices.

Representative transverse magnetization decay curves recorded before and after incubation are shown in **Fig. S6 (a-d)**. In the dry state, the overlaid profiles reveal a clear grouping of the extrudates into two distinct sets: formulations without Kleptose® (Aml, Aml-Dex, and Aml-DAc) and formulations containing Kleptose® (Aml-Kl, Aml-Kl-Dex, and Aml-Kl-DAc). This separation was consistently observed for both extrudate diameters (600 μm and 1000 μm). After incubation, the profiles largely overlapped across all formulations.

The relative contributions of the rigid and mobile phases before and after incubation are summarized in **Fig. S7 (a-d)**. Based on the fitted amplitudes, the fractional contribution of the T_2,2_ component (%) was determined for each formulation. Extrudates containing Kleptose® (Aml-Kl, Aml-Kl-Dex, and Aml-Kl-DAc) exhibited a distinct response to incubation, characterized by a slight increase in T_2,2_ relaxation times accompanied by a decrease in its relative contribution (Fig. 3, bottom). This trend was consistently observed for both 600 μm and 1000 μm extrudates. In contrast, extrudates without Kleptose® (Aml, Aml-Dex, and Aml-DAc) showed a decrease in T_2,2_ values after incubation, also accompanied by a reduction in the relative contribution of the T_2,2_ component. Consistently, the short relaxation component (T_2,1_) exhibited an increased relative contribution (%) after incubation for all formulations, together with a slight increase in T_2,1_ relaxation times (μs), indicating an overall shift toward more constrained proton environments within the hydrated matrices (Fig. 3, top).

Water penetration is a primary driver of molecular reorganization in starch-based matrices (Adamczyk et al., 2025; Sergeev et al., 2017). In semicrystalline systems with high amylose content, hydration does not necessarily result in homogeneous plasticization. Instead, absorbed water promotes rapid rearrangement and reassociation of polymer chains (Adamczyk et al., 2025; Colquhoun et al., 1995). While water initially enhances local mobility, it subsequently facilitates chain alignment and hydrogen-bond-driven ordering, a behavior commonly associated with early-stage retrogradation (Adamczyk et al., 2025; El Nokab et al., 2022).

The relaxometry data captures these hydration-induced rearrangements at the molecular scale (Adamczyk et al., 2025; Colquhoun et al., 1995). For cyclodextrin-free extrudates (Aml, Aml-Dex, and Aml-DAc), incubation resulted in a concurrent decrease in T_2,2_ relaxation times and in the relative contribution of the T_2,2_ component. This shift toward shorter T_2,2_ values and reduced mobile fractions is consistent with molecular stiffening rather than net matrix plasticization, as described in the literature. This interpretation is further supported by the increase in both the relative contribution and relaxation time of the short T_2,1_ component, indicating a redistribution of proton populations toward more constrained environments within the hydrated matrix.

This molecular response is consistent with the macroscopic hydration behavior of the extrudates. As shown in Fig. 2, all formulations exhibited rapid water uptake within the first hour of incubation. For cyclodextrin-free systems, hydration occurred without significant matrix erosion, indicating that the absorbed water was accommodated within the starch network. Under these conditions, water acts primarily as a reorganizing agent, facilitating chain rearrangement and partial ordering without matrix disintegration (Adamczyk et al., 2025; Colquhoun et al., 1995; Sergeev et al., 2017; El Nokab et al., 2022).

### 3.3 *In vitro* release assays

**Fig. 4:**
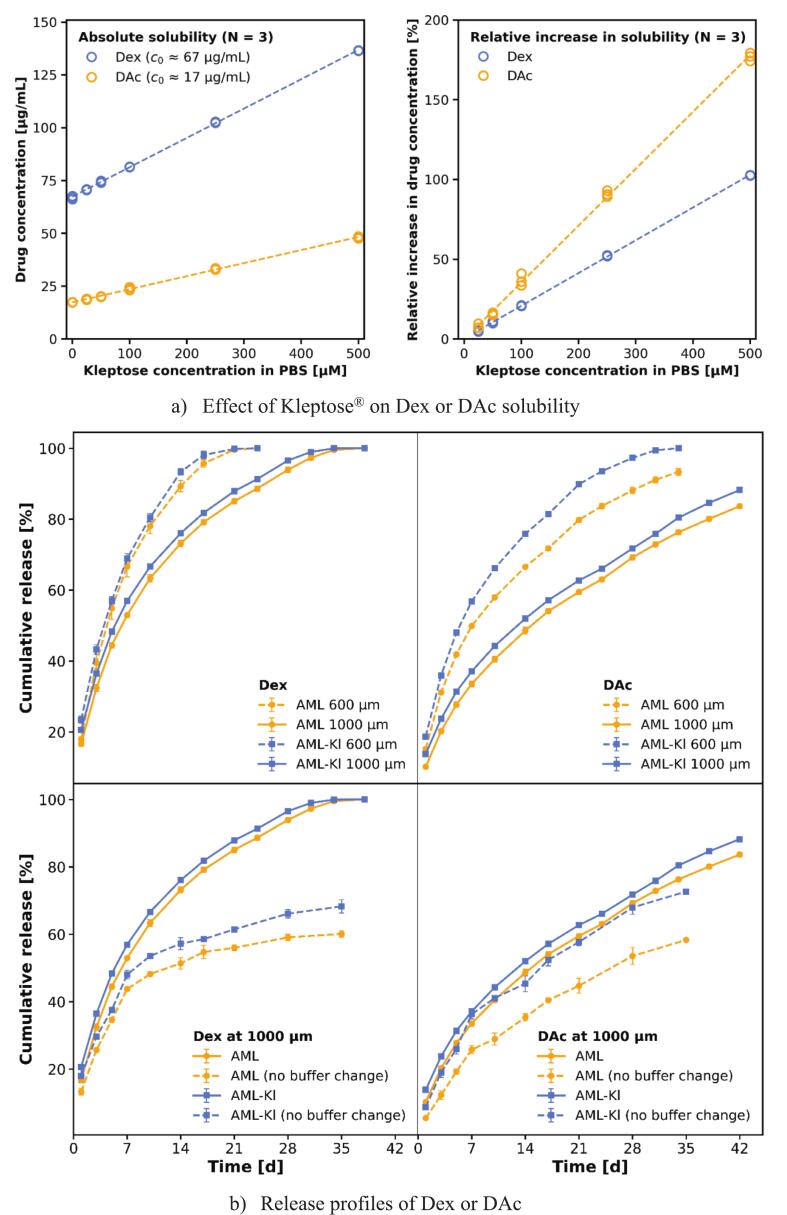
(a) Apparent solubility assays of dexamethasone and dexamethasone acetate under different concentrations of Kleptose®. (b) *In vitro* release profile of dexamethasone (top, left) and dexamethasone acetate (top, right) from 600 μm and 1000 μm starch-based extrudates. Comparison of the release curves of 1000 μm extrudates containing dexamethasone (bottom, left) and dexamethasone acetate (bottom, right) with and without buffer change.

Before evaluating drug release from the starch-based extrudates, an apparent solubility assessment of Dex and DAc was performed. The results are presented in Fig. 4 (a). The intrinsic solubilities of Dex (∼ 67 μg/mL) and DAc (∼ 17 μg/mL) in PBS at 37 °C are consistent with values previously reported in the literature (National Center for Biotechnology Information, 2024, Drug Bank, 2021, respectively).

The assays demonstrate that Kleptose® markedly enhances the apparent solubility of both corticosteroids in PBS in a concentration-dependent manner (Fig. 4, (a)). Over the investigated range (0–0.500 mM), a linear increase in drug concentration was observed for both compounds, with excellent correlation coefficients (Dex: R^2^= 0.998; DAc: R^2^= 0.999), indicating typical AL-type phase-solubility behavior (Higuchi and Connors, 1965). In absolute terms, the solubility of Dex increased from ∼ 67 μg /mL in PBS to ∼135 μg /mL at 0.500 mM Kleptose®, whereas DAc increased from ∼ 17 μg /mL to ∼ 48 μg /mL under the same conditions. When expressed as relative enhancement, this corresponds to an increase of approximately 100% for Dex and up to ∼180% for DAc at the highest cyclodextrin concentration. Notably, measurable solubility enhancement was already observed at the lowest concentrations tested (0.025–0.050 mM), demonstrating that even limited amounts of cyclodextrin can improve drug solubilization.

The *in vitro* release profiles of Dex and DAc are presented in Fig. 4 (b). The top panels show the release profiles obtained under sink conditions with periodic PBS replacement, whereas the bottom panels display the corresponding profiles under no buffer change conditions.

DAc is a well-established prodrug of dexamethasone (Derendorf et al., 1991; Sweetman, 2009). In the present study, the conversion of DAc to Dex was observed only after the prodrug was released into the PBS medium, but not in the solid extrudates. The hydrolysis followed an apparent first-order kinetic profile, as evidenced by the concentration-dependent decrease in DAc and the subsequent formation of Dex shown in **Fig. S8**. In addition to exhibiting a slower release profile than Dex, the conversion of the DAc prodrug into the active drug represents a secondary, time-dependent process. This behavior contributes to prolonging the effective duration of dexamethasone availability after release from the starch-based extrudates. Such behavior is consistent with the pharmacokinetic profile of corticosteroid ester formulations, in which lower solubility and the required conversion to the active drug contribute to sustained drug availability (Derendorf et al., 1991).

Under sink conditions, extrudate diameter was the primary factor governing the release rate, with 600 μm extrudates consistently releasing both drugs faster than 1000 μm systems. Comparison at 50% cumulative release highlights the effect of geometry while minimizing late-stage variability, confirming extrudate diameter as a dominant parameter controlling mass transport in starch-based matrices. In contrast, under no medium change (NMC) conditions, clear deviations from the standard release profiles were observed. The absence of medium renewal led to a progressive accumulation of released species in the surrounding medium, thereby altering the release behavior relative to sink conditions. While the release of Dex reached a plateau after approximately one week due to saturation of the buffer medium, DAc exhibited distinct release kinetics. Owing to the larger medium volume (50 mL vs. 15 mL) and the hydrolysis of DAc into the more soluble Dex, the release profile under NMC conditions more closely resembled that observed under sink conditions. In addition, the effect of Kleptose® was more pronounced under non-sink conditions, highlighting its role in enhancing drug solubilization.

The modulation of the release profiles is closely associated with the rapid hydration of the starch-based extrudates. Water uptake (Fig. 2), swelling (Fig. 2), and relaxometry assays (Fig. 3) showed that hydration occurs within the first 24 h of incubation. No delayed hydration phase was observed. Under these conditions, diffusion appears to be the predominant release mechanism for dexamethasone derivatives, which is consistent with established models for hydrated, non-eroding polymer matrices (Siepmann and Göpferich, 2001; Siepmann and Peppas, 2011) and with previous studies on starch-based extrudates exhibiting sustained diffusion-governed release behavior (Esfahani et al., 2022; Esfahani et al., 2023a). The faster release from the 600 μm extrudates can be attributed to their smaller cross-sectional dimension and higher surface-area-to-volume ratio compared to the 1000 μm extrudates. This geometrical configuration reduces the effective diffusion path length and enhances mass transport through the hydrated matrix, as described for cylindrical implantable systems governed by diffusion-controlled release (Iyer et al., 2006; Siepmann and Göpferich, 2001; Domsta et al., 2023).

Beyond hydration and geometry, the release profiles are also influenced by the presence of Kleptose®. As previously observed in Fig. 2, cyclodextrin is released from extrudates within the first 24 h of incubation. This early-stage dissolution contributes directly to the mass loss observed during matrix erosion. The release of Kleptose® likely results in the formation of microstructural voids within the starch matrix. Such void formation is a well-established consequence of the selective dissolution of hydrophilic excipients from solid polymer matrices, leading to increased porosity and enhanced water percolation (Bodmeier and Paeratakul, 1989; Siepmann and Göpferich, 2001; Siepmann and Peppas, 2011). These voids facilitate the penetration of aqueous media into the extrudates and promote the exposure and dissolution of dexamethasone crystals embedded within the hydrated matrix. Simultaneously, Kleptose® increases the apparent solubility of Dex and DAc in the surrounding medium, as shown in (Fig. 4). The incorporation of Kleptose® therefore operates through a coupled mechanism involving matrix modification and solubility enhancement.

Drug loads are presented in **Fig. S9 (a)**. All formulations exhibited drug loads exceeding 100%, which can be attributed to partial water evaporation during the extrusion process and the resulting apparent increase in drug concentration in the final extrudates. Despite this effect, content uniformity remained high across the formulations and corroborated the homogeneous drug distribution observed by Raman microscopy (Section 3.2.1, **Fig. S1 (d)**).

We decided to terminate the release assays after 42 days (6 weeks) since this time frame is likely the most relevant for the intended purpose of post-operative pain reduction. The recovered drug content after the release assays is shown in **Fig. S9 (b)**. The 600 μm extrudates released nearly 100% of both Dex and DAc in this time span. In contrast, complete drug release was not achieved for the DAc-containing formulations in the 1000 μm extrudates, which is consistent with the lower apparent solubility of DAc and the longer diffusion path associated with the larger extrudate diameter. The higher residual drug content observed mainly in the 1000 μm extrudates confirms that increasing the extrudate cross-sectional dimension results in a longer effective diffusion path through the hydrated matrix, thereby limiting complete drug release under the tested conditions. In addition, these experiments independently confirm the role of Kleptose® in promoting Dex and DAc release from starch-based extrudates. No evidence of drug degradation related to extrusion or sterilization was detected. This assessment is supported by the absence of additional chromatographic peaks and by the consistent PDA spectra associated with Dex and DAc (data not shown).

**Fig. S10** presents representative SEM micrographs of the cross-sections of the 1000 μm starch-based extrudates before and after 30 days of incubation in PBS. Before incubation, all samples exhibited compact and continuous cross-sections characterized by dense starch domains and the absence of macroscopic defects. No large cracks, pores, or voids were detected before incubation, confirming effective consolidation of the matrices during hot-melt extrusion.

After incubation, clear morphological differences were observed across all formulations. The incubated extrudates exhibited cracks and pores throughout the cross-sections, together with surface roughening and localized structural discontinuities. Elongated voids and channel-like features became apparent, suggesting possible formation of interconnected pathways along the extrudate structure. These features are consistent with progressive water penetration into the matrix and with the removal of soluble components during incubation. In Aml-Kl sample (**Figs. S10 (a)** and **(b)**), such features were present but remained relatively limited and less pronounced. In contrast, extrudates containing Dex or DAc, with or without Kleptose® (**Fig. S10 (c-j)**), exhibited more pronounced erosion patterns, including larger pores, interconnected voids, and deeper fissures within the starch matrix.

The release profiles were consistent with the complementary characterization results. Relaxometry, matrix erosion, SEM, and apparent solubility analyses collectively indicate that rapid matrix hydration, progressive structural changes, and the higher apparent solubility of Dex contribute to its faster release compared with DAc under both sink and non-sink conditions.

From a translational perspective, these starch-based extrudates are intended for local implantation at the surgical site immediately before wound closure. The release profiles observed in this study indicate sustained corticosteroid delivery throughout the early postoperative period, when inflammation and pain are most pronounced. Together with the biodegradable nature of the starch matrix, which eliminates the need for surgical implant removal after drug depletion, these findings support the potential clinical applicability of this formulation.

## 4 Conclusion

Cylindrical implantable extrudates were successfully produced by hot-melt extrusion using high-amylose starch. The process employed exclusively approved excipients and generated flexible matrices with homogeneous morphology. The resulting extrudates exhibited adequate mechanical robustness and structural integrity, confirming their suitability as a biodegradable platform for implantable controlled drug delivery.

All formulations exhibited rapid hydration without structural disintegration, accompanied by moderate swelling (∼30%) and limited matrix erosion. Formulations containing β-cyclodextrin showed early mass loss associated with cyclodextrin dissolution. ^1^H NMR relaxometry further indicated hydration-induced molecular reorganization within the starch matrix, with cyclodextrin-free extrudates exhibiting reduced chain mobility after incubation.

Drug release from extrudates was primarily governed by geometry. Smaller-diameter extrudates (600 μm) exhibited faster release due to their shorter diffusion path length and higher surface-area-to-volume ratio. In addition, the use of dexamethasone acetate introduced a secondary kinetic step through hydrolysis to dexamethasone, which may prolong the effective duration of drug availability.

Overall, starch-based extrudates enable rapid matrix hydration and sustained release of corticosteroids. These findings support their potential as biodegradable implants for localized anti-inflammatory therapy and postoperative pain management.

## CRediT authorship contribution statement

**Paulo Vitor França Lemos:** Writing – original draft, Validation, Methodology, Investigation, Formal analysis, Data curation, Conceptualization. **Julian Marcel Kunert:** Validation, Methodology, Formal analysis. **Marie-Luise Trutschel:** Validation, Methodology, Formal analysis. **Karsten Mäder:** Writing – review & editing, Supervision, Resources, Project administration, Methodology, Funding acquisition, Conceptualization.

## Funding

This work was financially supported by the 10.13039/100005156Alexander von Humboldt Foundation–10.13039/501100002322Coordenação de Aperfeiçoamento de Pessoal de Nível Superior (CAPES) agreement (grant 88887.674565/2022-00 and 88881.930122/2023-01).

## Declaration of competing interest

The authors declare that they have no known competing financial interests or personal relationships that could have appeared to influence the work reported in this paper.

## Data Availability

Data will be made available on request.
