# Supplementary material for: Implantable starch-based extrudates for controlled release of corticosteroids
Source: Int J Pharm X. 2026 Jul 29;12:100625. doi: 10.1016/j.ijpx.2026.100625 (PMC13453032; doi:10.1016/j.ijpx.2026.100625)
Supplement: Supplementary file 1 [file mmc1.docx]

**Implantable Starch-Based Extrudates for Controlled Release of Corticosteroids**

**Supplementary information (SI)**

Paulo Vitor França Lemos^1*^, Julian Marcel Kunert^1^, Marie-Luise Trutschel^1^, Karsten Mäder^1^

1 Institute of Pharmacy, Martin Luther University Halle-Wittenberg, Kurt-Mothes-Straße 3, 06120 Halle (Saale), Germany.

^*^ ^Corresponding author^

Table S1 – Summary of the preformulation tests to prepare the starch-based extrudates

| Sample | Amylo N-400^®^ (%), Dex or DAc (%), and Kleptose^®^ (%) | Diameter (μm) | Extrudate aspect |
| --- | --- | --- | --- |
| Aml | 100 | 600 or 1000 | Homog., flexible |
| Aml-Kl-5% | 95, 0, 5 | 600 or 1000 | Homog., flexible |
| Aml-Kl-10% | 90, 0, 10 | 600 or 1000 | Homog., flexible |
| Aml-Kl-20% | 80, 0, 20 | 600 or 1000 | Homog., brittle |
| Aml-Kl-30% | 70, 0, 30 | 600 or 1000 | Homog., brittle |
| Aml-Kl-40% | 60, 0, 40 | 600 or 1000 | Homog., very brittle |
| Kl-Dex-9% | 0, 9, 91 | 600 or 1000 | Homog., very brittle |
| Aml-Dex 9% | 91, 9, 0 | 600 or 1000 | Homog., flexible |
| Aml-DAc 9% | 91, 9,0 | 600 or 1000 | Homog., flexible |
| Aml-Kl 9% | 91, 0, 9 | 600 or 1000 | Homog., flexible |
| Aml-Kl-Dex 9% | 82, 9, 9 | 600 or 1000 | Homog., flexible |
| Aml-Kl-DAc 9% | 82, 9, 9 | 600 or 1000 | Homog., flexible |

|  |  |
| --- | --- |
| a) Aml-Kl-Dex 1000 µm | |
|  |  |
| b) Aml-Kl-DAc 1000 µm | |
|  |  |
| c) Aml-Kl 600 µm | |
|  | |
| d) Raman microscopy of Aml-Kl and Aml-Kl-Dex 1000 µm extrudates. | |

Figure S1 – Aspect of the flexible starch-based extrudates. (a) Aml-Kl-Dex 1000 µm, (b) Aml-Kl-DAc 1000 µm, (c) microscopic aspect of Aml-Kl 600 µm, and (d) content homogeneity of Aml-Kl and Aml-Kl-Dex 1000 µm extrudates evaluated by Raman microscopy. Raman mapping was performed using a 785 nm excitation laser in StreamLine mode over the 300–700 cm⁻¹ spectral range with a lateral step size of 3 µm to assess the spatial distribution of dexamethasone across the extrudate cross-sections. The representative Raman maps and spectra demonstrate the absence of localized drug-rich domains, indicating a homogeneous distribution of dexamethasone throughout the extrudate cross-section.

|  |  |  |
| --- | --- | --- |
| a) TG profiles of excipients, dexamethasone derivatives, physical mixtures and extrudates | b) DSC profiles of excipients, dexamethasone derivatives, physical mixtures and extrudates | c) Infrared spectra of Amylo N-400 and Kleptose |
|  |  |  |
| d) Infrared spectra of dexamethasone and dexamethasone acetate | e) Infrared spectra of a physical mixture and an extrudate containing dexamethasone acetate | f) XRD profiles of Amylo N-400 and Kleptose |
|  |  |  |
| g) XRD profiles of dexamethasone and dexamethasone acetate | h) XRD profiles of extrudates | i) XRD profiles of excipients, dexamethasone derivatives, physical mixtures and extrudates |

Figure S2 – Microstructure and thermal properties. (a) TG profiles of excipients, dexamethasone derivatives, physical mixtures and extrudates, and (b) DSC profiles of excipients, dexamethasone derivatives, physical mixtures and extrudates. (c) Infrared spectra of Amylo N-400^®^ and Kleptose^®^, (d) infrared spectra of dexamethasone and dexamethasone acetate, and (e) infrared spectra of a physical mixture and an extrudate containing dexamethasone acetate. (f) XRD profiles of Amylo N-400^®^ and Kleptose^®^, (g) XRD profiles of dexamethasone and dexamethasone acetate, (h) XRD profiles of extrudates, (i) XRD profiles of dexamethasone derivatives, physical mixtures, and extrudates.

Figure S3 – ^1^H NMR spectra of aqueous extracts obtained from starch-based extrudates without cyclodextrin (Aml), starch-based extrudates containing hydroxypropyl-β-cyclodextrin (Aml-Kl), and a reference solution of hydroxypropyl-β-cyclodextrin (Kleptose^®^), all prepared in 0.9% NaCl solution in D₂O (1 day, 37 °C). Characteristic β-cyclodextrin proton signals in the 3.4–3.8 ppm region are observed in the Kleptose reference and in the Aml–Kl extract, but are absent in the Aml sample. This experiment confirms the identity of the compound released during the gravimetric matrix erosion assay.

|  |  |
| --- | --- |
| a) Aml before incubation | b) Aml after incubation |
|  |  |
| c) Aml-Dex before incubation | d) Aml-Dex after incubation |
|  |  |
| e) Aml-DAc before incubation | f) Aml-DAc after incubation |
|  |  |
| g) Aml-Kl before incubation | h) Aml-Kl after incubation |
|  |  |
| i) Aml-Kl-Dex before incubation | j) Aml-Kl-Dex after incubation |
|  |  |
| k) Aml-Kl-DAc before incubation | l) Aml-Kl-DAc after incubation |

Figure S4 – Relaxometry assays. Curve fittings of the 600 µm extrudates. The Aml (a-b), Aml-Dex (c-d), Aml-DAc (e-f), Aml-Kl (g-h), Aml-Kl-Dex (i-j), and Aml-Kl-DAc (k-l) extrudates were evaluated before and after incubation in 0.9% NaCl solution in D_2_O (1 hour, 37 ºC).

|  |  |
| --- | --- |
| a) Aml before incubation | b) Aml after incubation |
|  |  |
| c) Aml-Dex before incubation | d) Aml-Dex after incubation |
|  |  |
| e) Aml-DAc before incubation | f) Aml-DAc after incubation |
|  |  |
| g) Aml-Kl before incubation | h) Aml-Kl after incubation |
|  |  |
| i) Aml-Kl-Dex before incubation | j) Aml-Kl-Dex after incubation |
|  |  |
| k) Aml-Kl-DAc before incubation | l) Aml-Kl-DAc after incubation |

Figure S5 – Relaxometry assays. Typical curve fittings of the 1000 µm extrudates. The Aml (a-b), Aml-Dex (c-d), Aml-DAc (e-f), Aml-Kl (g-h), Aml-Kl-Dex (i-j), and Aml-Kl-DAc (k-l) extrudates were evaluated before and after incubation in 0.9% NaCl solution in D_2_O (1 hour, 37 ºC).

|  |  |
| --- | --- |
| a) Extrudates 600 µm before incubation | b) Extrudates 600 µm after incubation |
|  |  |
| c) Extrudates 1000 µm before incubation | d) Extrudates 1000 µm after incubation |

Figure S6 – Relaxometry assays. Transverse magnetization decay for 600 µm extrudates (a) before and (b) after incubation and 1000 µm extrudates (c) before and (d) after incubation. The extrudates were evaluated before and after incubation in 0.9% NaCl solution in D_2_O (1 hour, 37 ºC).

|  |  |
| --- | --- |
| a) Extrudates 600 µm before incubation | b) Extrudates 600 µm after incubation |
|  |  |
| c) Extrudates 1000 µm before incubation | d) Extrudates 1000 µm after incubation |

Figure S7 – Contributions of the mobile and rigid phases of the extrudates 600 µm (a) before and (b) after incubation and extrudates 1000 µm (c) before and (d) after incubation calculated by fitting mono- and double exponential decay curves. The extrudates were evaluated before and after incubation in 0.9% NaCl solution in D_2_O (1 hour, 37 ºC)

Figure S8– Dexamethasone acetate degradation assessment in PBS at 37 ºC

a) Drug load of the dexamethasone and dexamethasone acetate extrudates.

b) Recovered content after the release assays

Figure S9. (a) Drug load of the extrudates and (b) recovered drug content after the release assays.

|  |  |  |  |
| --- | --- | --- | --- |
| a) Aml-Kl before incubation | | b) Aml-Kl after incubation | |
|  |  |  |  |
| c) Aml-Kl-Dex before incubation | | d) Aml-Kl-Dex after incubation | |
|  |  |  |  |
| e) Aml-Dex before incubation | | f) Aml-Dex after incubation | |
|  |  |  |  |
| g) Aml-Kl-DAc before incubation | | h) Aml-Kl-DAc after incubation | |
|  |  |  |  |
| i) Aml-DAc before incubation | | j) Aml-DAc after incubation | |

Figure S10 – SEM micrographs of the 1000 μm starch-based extrudates before and after incubation
